# Association between adherence to the Dietary Approaches to Stop Hypertension diet and serum uric acid

**DOI:** 10.1038/s41598-023-31762-x

**Published:** 2023-04-18

**Authors:** Jie Feng, Yuchai Huang, Haozhuo Wang, Chao Wang, Hongbin Xu, Pan Ke, Yan He, Qingfeng Tian, Shiyi Cao, Zuxun Lu

**Affiliations:** 1 Department of Social Medicine and Health Management, School of Public Health, Tongji Medical College, Huazhong University of Science and Technology, Wuhan, China; 2 Health Management Center, Tongji Hospital, Tongji Medical College, Huazhong University of Science and Technology, Wuhan, Hubei China; 3 School of Public Health and Management, Chongqing Medical University, Chongqing, China; 4 School of Public Health, Wuhan University, Wuhan, Hubei China; 5 School of Public Health, Zhengzhou University, Zhengzhou, Henan China

**Keywords:** Diseases, Health care, Risk factors

## Abstract

To explore the relationship between Dietary Approaches to Stop Hypertension (DASH) diet and serum uric acid (SUA) levels among the Chinese adult population, and verify the mediating effect of BMI between DASH diet and SUA levels. A total of 1125 adults were investigated using a self-administered food frequency questionnaire. SUA levels were determined by uricase colorimetry. The total DASH score ranged from 9 to 72. The relationship between the DASH diet and SUA levels was examined by multiple adjusted regression analysis. Method of Bootstrap was used to test the mediation effect of BMI in the correlation of the DASH diet and SUA levels. After multivariable adjustment, there was a significant linear relationship between the DASH diet and SUA (*P* < 0.001). Compared with the lowest group, SUA of participants in group of highest DASH diet score decreased by 34.907 (95% CI − 52.227, − 17.588; *P* trend < 0.001) μmol/L. The association between the DASH diet scores and SUA levels was partly mediated by BMI (− 0.26, Bootstrap 95% CI − 0.49, − 0.07), with 10.53% of the total effect being mediated. Adopting the DASH diet might be helpful in reducing SUA level, and the effect might be partly mediated by BMI.

## Introduction

An elevated level of serum uric acid (SUA) is a critical cause of gout^1–3^. It is estimated that 21% of the population suffers from hyperuricemia and 1–4% from gout^4^. Studies have shown that elevated SUA levels are also closely related to cardiovascular diseases (CVD) such as hypertension, obesity, and dyslipidemia^5–7^. Furthermore, studies have confirmed that SUA levels are determined by certain uncontrollable factors, including genetic or hereditary causes^8,9^.Besides, it could be also influenced by food intake^10^, a factor that could be controlled.

In recent years, the importance of overall dietary intake to prevent excessive SUA levels has gradually increased. A study from the USA showed that the SUA levels of subjects decreased from 7.7 mg/dL (SD = 1.7 mg/dL) to 5.4 mg/dL (SD = 2.7 mg/dL) after two weeks on a Purine-Low Diet^11^. Another study from Greece reported that the subjects’ SUA levels had dropped from the baseline of 9.12–6.2 mg/dL by complying with the Mediterranean-Style diet for 8 weeks^12^. However, the current experiments, which aimed to explore the association between dietary patterns and SUA levels, mainly focused on Western Dietary Pattern, Mediterranean-Style diet and so on^13–17^, while there is little epidemiological evidence on the association between the Dietary Approaches to Stop Hypertension (DASH) diet and SUA levels.

Residents in coastal cities in eastern and southern China (e.g. Shenzhen) have greater access to seafood including shellfish, shrimp and crab that are high in purines, which may be related to the elevated SUA levels^18,19^.

The DASH diet advocates a high intake of whole grains, fruits, vegetables, and low-fat dairy products, a low intake of saturated fat, red meat, processed meat, sweets and sodium^20–24^. DASH diet may contribute to control weight and SUA levels, as well may be beneficial in the prevention and management of CVD risk^25,26^. Up to now, certain international studies have successively demonstrated the relationship between the DASH diet and SUA levels. Several randomized controlled trials have also shown that the DASH diet can significantly lower SUA levels in individuals with hyperuricemia^27,28^. Nevertheless, so far, relevant studies on the correlation between the DASH diet and SUA levels, have mainly carried out by western countries such as the USA, while there is little direct evidence that DASH diet can reduce SUA levels in China and developing countries.

As was proved that adopting the DASH diet is beneficial of reducing body weight and then maintaining a healthy body weight, especially for overweight and obese people^29^, and the SUA levels are highly associated with overweight, obesity and other risk factors^30–32^. Therefore, it can be hypothesized that BMI may be an intermediate variable in the association between the DASH diet and SUA levels, and that adherence to the DASH diet could, to a certain extent, reduce the body weight of patients, which decreases their SUA levels in turn.

We aim to understand the current status of the DASH diet and SUA levels on the population in China, and explore the association between the DASH diet and SUA level. Till now, there is no study reporting whether BMI mediates the DASH diet and SUA levels. Therefore, we further identify the mediating effect of BMI between the DASH diet and SUA levels.

## Methods

### Study design and sample selection

A cross-sectional survey on the risk assessment about hyperuricemia and gout of community residents was conducted in three different community health service centers in Shenzhen, China, from April 2019 to September 2020. Convenience sampling was adopted to complete this survey, through on-site or online channels to recruit participants-sending free SUA test notifications to participants through family physicians, or recruiting participants in community health service centers’ outpatient clinics. Participants who were desire to have a measurement of SUA would be tested for free and included in the study after informed consent.

Our research is a single-blinded cross-sectional study. After completing the blood collection and questionnaire filling, we would not feedback about the results of SUA levels and questionnaire filling to the participants. All data were input into the proprietary computer and accessible to named study personnel only, and only in forms without identifying personal identification (such as the participants’ names, telephone numbers) of any kind other than unique study ID number. In addition, the data was encrypted to ensure that it could not be copied or distributed over the Internet.

This study adhered to the STROBE checklist indicating the page/line numbers of our manuscript where the relevant information can be found. (Supplementary appendix 1).

### Inclusion and exclusion criteria

Participants selected for this study had to meet the following criteria: (1) permanent residents aged 18 and above in Shenzhen. Permanent residents refer to those who were born before September 1, 2001, and have lived in Shenzhen for more than 6 months in the past 12 months; (2) Participants were required to fasting for 8 or more hours before testing; (3) normal cognitive and language expression abilities and no verbal communication barriers; and (4) sufficiently informed of the study’s purpose and content, voluntarily choosing to participate.

Exclusion criteria: (1) Participants who did not complete the questionnaires or who refused to sign the informed consent; (2) Pregnant or breastfeeding women and patients of disease, such as severe mental illness, infectious diseases, and allomnesia were excluded.

### Ethical approval

This study was approved by the Research Ethics Committee of the Tongji Medical College, Huazhong University of Science and Technology, Wuhan, China, and performed in accordance with the principles of the Declaration of Helsinki. All participants had signed consent form before filling out the questionnaire, and personal information of all participants was kept confidential.

### Instrument and measurement

The questionnaire included the following aspects: the demographic characteristics, lifestyles, history of diseases, family history of gout (yes, no) and dietary habits. The demographic characteristics included gender (female, male), age (years, continuous), BMI (kg/m^2^, continuous), marital status (married, not married), education level (college degree less, college degree or higher), occupation (medical staffs, not medical staffs). The lifestyles included smoking (yes, no), alcohol drinking (yes, no), uric-acid-lowering drug intake (yes, no). History of diseases included history of hypertension (yes, no), history of diabetes (yes, no), history of hyperuricemia (yes, no), history of gout (yes, no). The dietary habits included 18 types of food, refined grain, coarse grain, fresh fruits, fruit products, fresh vegetables, pickles, whole milk, low-fat milk, red meats, white meats, animal offal, seafood, processed meats, legumes and their products, nuts, sweet drinks, desserts and smoked products. A nine-point scale was adopted to record the frequency of taking above-mentioned food^33^. (The details about the questionnaire are shown in Supplementary appendix 2.).

#### Calculating the DASH diet score

In this study, the DASH diet score is calculated based on the intake frequency of eight food components^23^. The DASH diet mode proposes individual intake recommendations for each food components, that is, high intake of whole grains, fruits, vegetables, nuts and legumes, low fat dairy products, and less intake of sodium, sweetened beverages, red meats and processed meats. Analysis in this study focuses on the perspective of the willingness and propensity of food intake, thus avoiding bias caused by inaccurate food intake estimates, where the sodium intake is substituted by the intake frequency of salty pickles and smoked products, nuts and beans classified as the same group, red meats and processed meats classified as the same group, every component is weighted equally during dietary patterns evaluation^23,34^. The participants reported frequency of intake using the following response categories for fruits, vegetables, legumes, nuts, low-fat milk and whole grains, ranging from 1 (less than once per month), 2 (1–3 times per month), 3 (once per week), 4 (1–3 times per week), 5 (4–6 times per week), 6 (once per day), 7 (2–3 times per day), 8 (4–5 times per day) to 9 (more than 5 times per day). Sodium, sugary sweetened beverages, and red and processed meats are scored in reverse, ranging from 9 (less than once per month) to 1 (more than 5 times per day)^35^. The whole questionnaire yields a DASH diet score from 9 to 72, and participants with higher DASH scores were more likely to adopt the DASH diet. The Cronbach’s alpha value was 0.784, indicating that food frequency questionnaire had good consistency, and the Kaiser–Meyer–Olkin (KMO) value (0.846) and Bartlett’s test of sphericity value (χ^2^ = 4641.197, *P* < 0.001) indicated good validity.

#### Ascertainment of serum uric acid

SUA levels were measured by the uricase colorimetry method^36^. Participants were required to fasting for 8 or more hours before testing, then serum specimens were collected and immediately delivered to Fuyong People’s Hospital for analysis. The absorbance was measured at 37 °C and a wavelength of 490 nm, and internal quality control was performed during the measurement process. SUA was detected by Hitachi 7600 automatic biochemical analyzer with the detection kit (Shenzhen Mindray Bio-Medical Electronics Co., Ltd., Shenzhen, China) and comparison kit (Jiaxing Botai Biotechnology Development Co., Ltd., Jiaxing, China) used the relative deviation between them does not exceed 15%. Elevated SUA was diagnosed as SUA levels above 420 μmol/L in males or 360 μmol/L in females^37^.

### Statistical analysis

In this study, analyses were performed using Statistical Package for Social Sciences (SPSS, Inc., Chicago, III, Version 23.0). Descriptive analyses were performed to analyze characteristics among participants. Specifically, means and standard deviations (SD) for continuous variables, frequencies and percentages for categorical variables were reported separately in our research. The relationship between the DASH diet and SUA levels was examined by multiple adjusted regression analysis. Hierarchical multiple regression analysis was performed to examine the interaction of stratified variables with DASH scores. To test the stability of the association between the DASH diet and SUA levels, we conducted a sensitivity analysis by excluding individuals who had been diagnosed with common chronic diseases and family history of the diseases, SUA outliers (> $\overline{x } \pm$3SD), and uric-acid-lowering drug intake. Finally, method of Bootstrap was used to test the mediation effect of body mass index (BMI) in the correlation of the DASH diet and SUA levels. All statistical tests were two-tailed, and *P* value < 0.05 was considered to be statistically significant.

## Results

### The SUA levels of participants

A total of 1125 participants were involved in this study, including 673 males (59.8%) and 452 females (54.3%). Participants’ mean SUA levels were (388.2 ± 142.3) μmol/L, of which the mean SUA for males were (449.0 ± 165.0) μmol/L, and (280.0 ± 122.0) μmol/L for females. Among 1125 adults, 516 had elevated SUA levels, including 419 males(81.2%) and 97 females(18.8%) (Table 1).

**Table 1:** The SUA levels of participants.

Characteristic	Total	Elevated SUA	Normal SUA	*P*
	n = 1125	n = 516	n = 609	
Age	40 ± 16.0	42 ± 12.0	40 ± 11.0	0.043
BMI	23.7 ± 4.7	25.1 ± 3.5	22.7 ± 3.3	< 0.001
Gender				< 0.001
Males	673 (59.8)	419 (81.2)	254 (41.7)	
Females	452 (40.2)	97 (18.8)	355 (58.3)	
Marital status				0.028
Married	929 (82.6)	440 (85.3)	489 (80.3)	
Not married	196 (17.4)	76 (14.7)	120 (19.7)	
Occupation				< 0.001
Medical staffs	253 (22.5)	37 (7.2)	216 (35.5)	
Not medical staffs	872 (77.5)	479 (92.8)	393 (64.5)	
Education level				< 0.001
College degree less	801 (71.2)	444 (86.0)	252 (41.4)	
College degree or higher	324 (28.8)	72 (14.0)	357 (58.6)	
Smoking				< 0.001
Yes	260 (23.1)	174 (33.7)	86 (14.1)	
No	865 (76.9)	342 (66.3)	523 (85.9)	
Alcohol drinking				< 0.001
Yes	257 (22.8)	171 (33.1)	86 (14.1)	
No	868 (77.2)	345 (66.9)	523 (85.9)	
History of hypertension				< 0.001
Yes	292 (26.0)	190 (36.8)	102 (16.7)	
No	833 (74.0)	326 (63.2)	507 (83.3)	
History of diabetes				0.261
Yes	78 (6.9)	31 (6.0)	47 (7.7)	
No	1047 (93.1)	485 (94.0)	562 (92.3)	
History of hyperuricemia				0.635
Yes	258 (22.9)	115 (22.3)	143 (23.5)	
No	867 (77.1)	401 (77.7)	466 (76.5)	
History of gout				0.032
Yes	152 (13.5)	82 (15.9)	70 (11.5)	
No	973 (86.5)	434 (84.1)	539 (88.5)	
Family history of gout				0.297
Yes	53 (4.7)	28 (5.4)	25 (4.1)	
No	1072 (95.3)	488 (94.6)	584 (95.9)	
Uric-acid-lowering drug intake				< 0.001
Yes	100 (8.9)	89 (17.2)	11 (1.8)	
No	1025 (91.1)	427 (82.8)	598 (98.2)	

Values are the number (%) or the mean (± SE) unless indicated otherwise.

It was suggested that there were differences in distributions between the group with elevated SUA and the group with normal SUA (*P* < 0.05), by age, BMI, gender, marital status, occupation, education level, smoking, alcohol drinking, history of hypertension, history of gout, and uric-acid-lowering drug intake (Table 1).

### Characteristics of study participants across quartiles of DASH diet score

A total of 1125 adults were included for analysis. The average value of the DASH diet score was 37.7 ± 4.9. The mean SUA levels of participants was 435.0 ± 146.1 μmol/L in group of the lowest the DASH diet scores, was 334.4 ± 125.5 μmol/L in group of the highest the DASH diet scores. The mean BMI of participants was 22.8 ± 4.7 kg/m^2^ in group of the highest the DASH diet scores, was 24.6 ± 4.4 in group of the lowest the DASH diet scores (Table 2).

**Table 2:** Characteristics of participants according to DASH diet score.

Factor	Quartiles of DASH diet score				*P*
	Quartile 1 (n = 296)	Quartile 2 (n = 284)	Quartile 3 (n = 282)	Quartile 4 (n = 263)	
Gender (males)	226 (76.4)	186 (65.5)	152 (53.9)	109 (41.4)	< 0.001
Age (years)	37.1 ± 14.5	40.6 ± 16.9	41.6 ± 15.7	38.9 ± 14.4	< 0.001
BMI (kg/m^2^)	24.6 ± 4.4	24.0 ± 3.9	23.2 ± 5.0	22.8 ± 4.7	< 0.001
SUA (μmol/L)	435.0 ± 146.1	393.9 ± 134.3	383.5 ± 143.7	334.4 ± 125.5	< 0.001
Marital status (yes)	233 (78.7)	233 (82.0)	239 (84.8)	224 (85.2)	0.152
Occupation (medical staffs)	45 (15.2)	56 (19.7)	71 (25.2)	81 (30.8)	< 0.001
Education (college degree less)	230 (77.7)	210 (73.9)	198 (70.2)	163 (62.0)	< 0.001
Smoking (yes)	103 (34.8)	66 (23.2)	54 (19.1)	37 (14.1)	< 0.001
Alcohol drinking (yes)	93 (31.4)	64 (22.5)	57 (20.2)	43 (16.3)	< 0.001
History of hypertension (yes)	73 (24.7)	86 (30.3)	95 (33.7)	38 (14.4)	< 0.001
History of diabetes (yes)	14 (4.7)	17 (6.0)	23 (8.2)	24 (9.1)	0.155
History of hyperuricemia (yes)	64 (21.6)	59 (20.8)	83 (29.4)	52 (19.8)	0.026
History of gout (yes)	63 (21.3)	38 (13.4)	26 (9.2)	25 (9.5)	< 0.001
Family history of gout (yes)	19 (6.4)	14 (4.9)	8 (2.8)	12 (4.6)	0.243
Uric-acid-lowering drug intake (yes)	39 (13.2)	28 (9.9)	16 (5.6)	17 (6.5)	0.006

Values are the number (%) or the mean (± SE) unless indicated otherwise.

### The results of multivariate linear regression analysis of the DASH diet score associated with SUA levels

After multi-variable adjustment, there was a significantly linear relationship between the DASH diet scores and SUA levels (*P* < 0.001). Every unit increase in the DASH diet score was associated with a 2.199(95% CI − 3.446, − 0.951; *P* < 0.001) μmol/L decrease in SUA. SUA levels of participants in group of highest the DASH diet scores decreased by 34.907 (95% CI − 52.227, − 17.588; *P*_trend_ < 0.001) μmol/L, when compared with the lowest group (Table 3).

**Table 3:** Multivariate linear regression analysis of DASH diet score associated with SUA levels.

Model	Quartiles of DASH diet score					Per increase	*P*
	Q1	Q2	Q3	Q4	*P*_trend_		
Model 1	Ref.	− 41.086 (− 63.569, − 18.604)	− 51.417 (− 73.940, − 28.894)	− 100.549 (− 123.485, − 77.613)	< 0.001	− 7.391 (− 9.024, − 5.759)	< 0.001
Model 2	Ref.	− 21.422 (− 39.581, − 3.264)	− 10.948 (− 29.503, 7.608)	− 34.212 (− 53.485, − 14.939)	0.003	− 2.217 (− 3.604, − 0.830)	0.002
Model 3	Ref.	− 21.077 (− 39.225, − 2.928)	− 10.261 (− 28.857, 8.334)	− 30.912 (− 50.198, − 11.626)	0.005	− 1.994 (− 3.383, − 0.605)	0.005
Model 4	Ref.	− 19.241 (− 35.656, − 2.826)	− 17.083 (− 34.050, − 0.116)	− 37.594 (− 55.128, − 20.060)	< 0.001	− 2.392 (− 3.655, − 1.129)	< 0.001
Model 5	Ref.	− 18.043 (− 34.263, − 1.824)	− 14.331 (− 31.093, 2.430)	− 34.907 (− 52.227, − 17.588)	< 0.001	− 2.199 (− 3.446, − 0.951)	< 0.001

Model 1: the primary model.

Model 2: Adjusted for gender, age, married status, occupation, education level.

Model 3: Based on Model 2, Adjusted for smoking, alcohol drinking, history of hypertension, history of diabetes.

Model 4: Based on Model 3, Adjusted for history of hyperuricemia, history of gout, family history of gout, uric-acid-lowering drug intake.

Model 5: Based on Model 4, Adjusted for BMI.

### The results of sensitivity analysis of the association between the DASH diet score and SUA levels

In the sensitivity analyses, exclusion of the participants with self-reported chronic diseases, family history of diseases, the extreme values of SUA and uric-acid-lowering drug intake did not materially change the observed the association between DASH diet score and SUA levels (*P* for trend < 0.05 for all) (Table 4).

**Table 4:** Sensitivity analysis of the association between DASH diet score and SUA levels.

Model	Quartiles of DASH diet score					Per increase	*P*
	Q1	Q2	Q3	Q4	*P*_trend_		
A	Ref.	− 16.449 (− 41.909, 9.011)	− 4.742 (− 30.626, 21.142)	− 34.761 (− 59.667, − 9.856)	0.015	− 2.401 (− 4.137, − 0.665)	0.007
B	Ref.	− 13.755 (− 40.197, 12.686)	− 24.578 (− 52.257, 3.100)	− 32.150 (− 59.131, − 5.168)	0.015	− 1.952 (− 3.767, − 0.138)	0.035
C	Ref.	− 22.200 (− 38.037, − 6.363)	− 21.300 (− 37.642, − 4.957)	− 38.822 (− 55.661, − 21.984)	< 0.001	− 2.415 (− 3.628, − 1.203)	< 0.001
D	Ref.	− 22.131 (− 38.732, − 5.531)	− 19.265 (− 36.169, − 2.361)	− 32.845 (− 50.350, − 15.341)	0.001	− 2.104 (− 3.349, − 0.859)	0.001

Model A: Excluding participants with history of diabetes, history of hypertension, history of hyperuricemia and gout, adjusted for gender, age, BMI, married status, occupation, education level, smoking, alcohol drinking, family history of gout and uric-acid-lowering drug intake.

Model B: Excluding participants with history of diabetes, history of hypertension, history of hyperuricemia, history of gout, family history of gout, adjusted for gender, age, BMI, married status, occupation, education level, smoking, alcohol drinking, and uric-acid-lowering drug intake.

Model C: Excluding participants with the extreme values of SUA, adjusted for gender, age, BMI, married status, occupation, education level, smoking, alcohol drinking, history of diabetes, history of hypertension, history of hyperuricemia, history of gout, family history of gout and uric-acid-lowering drug intake.

Model D: Excluding participants with uric-acid-lowering drug intake, adjusted for gender, age, BMI, married status, occupation, education level, smoking, alcohol drinking, history of diabetes, history of hypertension, history of hyperuricemia, history of gout, family history of gout.

### The results of stratified analysis of the association between DASH diet score and SUA levels

Results of subgroup analysis showed that the relationship between the DASH scores and SUA levels was significant in non-smoking, non-hypertension and non-hyperuricemia (*P* < 0.05), and was non-significant in smokers and those with history of hypertension or hyperuricemia (*P* < 0.05). For non-smoking participants, every unit increase in the DASH diet score was associated with a 2.916(95% CI − 4.280, − 1.552, *P* < 0.001) μmol/L decrease in SUA. For non-hypertension participants, every unit increase in the DASH diet score was associated with a 2.017(95% CI − 3.375, − 0.659, *P* = 0.004) μmol/L. For non-hyperuricemia participants, every unit increase in the DASH diet score was associated with a 2.135(95% CI − 3.502, − 0.769, *P* < 0.001). (Table 5).

**Table 5:** Stratified analysis of the association between the DASH diet score and SUA levels.

Model	Quartiles of DASH score					Per increase	*P*	*P* for interaction
	Q1	Q2	Q3	Q4	*P*_*trend*_			
Gender^a^								
Male	Ref.	− 20.759 (− 41.517, − 0.001)	− 10.960 (− 33.319, 11.398)	− 34.573 (− 59.674, − 9.473)	0.019	− 1.515 (− 3.313, 0.283)	0.098	0.287
Female	Ref.	− 10.767 (− 36.158, 14.623)	− 19.427 (− 43.461, 4.607)	− 34.119 (− 57.470, − 10.769)	0.002	− 2.487 (− 4.081, − 0.892)	0.002	
BMI^b^								
BMI ≥ 25 kg/m^2^	Ref.	− 28.827 (− 52.285, − 5.370)	− 16.669 (− 41.975, 8.638)	− 40.244 (− 67.435, − 13.052)	0.012	− 2.881 (− 4.882, − 0.881)	0.005	0.419
BMI < 25 kg/m^2^	Ref.	− 4.492 (− 27.065, 18.081)	− 12.970 (− 35.178, 9.237)	− 32.571 (− 54.931, − 10.210)	0.003	− 1.840 (− 3.397, − 0.283)	0.021	
Marital status^c^								
Married	Ref.	− 21.178 (− 38.569, − 3.788)	− 21.087 (− 38.819, − 3.355)	− 35.773 (− 54.293, − 17.253)	< 0.001	− 2.225 (− 3.601, − 0.849)	0.002	0.493
Not married	Ref.	− 11.981 (− 52.497, 28.534)	2.841 (− 41.420, 47.101)	− 46.035 (− 91.878, − 0.192)	0.116	− 2.406 (− 5.254, 0.443)	0.097	
Occupation^d^								
Medical staffs	Ref.	− 21.932 (− 53.993, 10.128)	− 22.229 (− 53.097, 8.639)	− 43.159 (− 73.262, − 13.056)	0.007	− 2.219 (− 4.123, − 0.315)	0.023	0.383
Not medical staffs	Ref.	− 16.759 (− 35.445, 1.928)	− 16.680 (− 36.293, 2.933)	− 35.298 (− 55.877, − 14.719)	0.002	− 2.351 (− 3.872, − 0.830)	0.002	
Education level^e^								
College degree less	Ref.	− 12.442 (− 32.235, 7.350)	− 10.129 (− 30.818, 10.560)	− 28.757 (− 51.022, − 6.493)	0.022	− 1.853 (− 3.490, − 0.215)	0.027	0.081
College degree or higher	Ref.	− 30.425 (− 58.023, − 2.828)	− 31.599 (− 58.612, − 4.585)	− 48.568 (− 75.013, − 22.124)	0.001	− 2.301 (− 4.080, − 0.523)	0.011	
Smoking^f^								
Yes	Ref.	6.068 (− 29.830, 41.966)	2.998 (− 35.542, 41.538)	− 28.935 (− 72.587, 14.717)	0.318	0.148 (− 2.853, 3.150)	0.922	0.038*
No	Ref.	− 26.373 (− 44.736, − 8.011)	− 22.310 (− 40.937, − 3.684)	− 42.369 (− 61.311, − 23.426)	< 0.001	− 2.916 (− 4.280, − 1.552)	< 0.001	
Alcohol drinking^g^								
Yes	Ref.	12.952 (− 27.148, 53.051)	− 5.610 (− 47.704, 36.484)	− 15.091 (− 60.309, 30.128)	0.461	− 0.278 (− 3.571, 3.014)	0.868	0.170
No	Ref.	− 27.982 (− 45.592, − 10.372)	− 20.957 (− 38.972, − 2.941)	− 44.328 (− 62.773, − 25.884)	< 0.001	− 2.707 (− 4.020, − 1.395)	< 0.001	
History of hypertension^h^								
Yes	Ref.	− 42.628 (− 74.272, − 10.984)	− 35.303 (− 66.768, − 3.838)	− 23.901 (− 64.017, 16.214)	0.148	− 2.652 (− 5.786, 0.482)	0.097	0.009*
No	Ref.	− 10.097 (− 29.012, 8.817)	− 7.656 (− 27.479, 12.167)	− 34.666 (− 53.912, − 15.421)	0.001	− 2.017 (− 3.375, − 0.659)	0.004	
History of diabetes^i^								
Yes	Ref.	14.962 (− 58.904, 88.829)	19.220 (− 43.753, 82.192)	− 5.665 (− 77.948, 66.617)	0.910	− 1.048 (− 5.733, 3.636)	0.656	0.089
No	Ref.	− 21.188 (− 37.796, − 4.480)	− 17.022 (− 34.377, 0.333)	− 39.290 (− 57.070, − 21.509)	< 0.001	− 2.361 (− 3.642, − 1.081)	< 0.001	
History of hyperuricemia^j^								
Yes	Ref.	− 9.414 (− 45.162, 26.333)	10.978 (− 23.697, 45.654)	− 22.772 (− 61.443, 15.899)	0.535	− 1.809 (− 4.802, 1.184)	0.235	0.013*
No	Ref.	− 18.910 (− 36.947, − 0.873)	− 20.983 (− 40.171, − 1.795)	− 36.729 (− 56.004, − 17.454)	< 0.001	− 2.135 (− 3.502, − 0.769)	0.002	
History of gout^k^								
Yes	Ref.	− 5.472 (− 56.267, 45.322)	46.604 (− 15.791, 108.999)	− 48.297 (− 110.619, 14.025)	0.415	− 0.969 (− 5.780, 3.843)	0.691	0.113
No	Ref.	− 19.560 (− 36.368, − 2.753)	− 24.217 (− 41.258, − 7.175)	− 36.100 (− 53.697, − 18.503)	< 0.001	− 2.417 (− 3.671, − 1.164)	< 0.001	
Family history of gout^l^								
Yes	Ref.	− 22.604 (− 93.284, 48.076)	− 21.780 (− 122.976,79.416)	− 65.433 (− 162.045, 311.179)	0.188	− 3.040 (− 10.476, 4.396)	0.412	0.258
No	Ref.	− 18.935 (− 35.446, − 2.423)	− 18.309 (− 35.231, − 1.387)	− 35.646 (− 53.118, − 18.174)	< 0.001	− 2.188 (− 3.437, − 0.939)	0.001	
Uric-acid-lowering drug intake^m^								
Yes	Ref.	22.533 (− 44.319, 89.386)	65.544 (− 13.427, 144.516)	− 67.788 (− 151.392, 15.817)	0.582	− 1.125 (− 7.817, 5.567)	0.739	0.395
No	Ref.	− 23.104 (− 39.672, − 6.535)	− 20.003 (− 36.859, − 3.147)	− 34.501 (− 51.976, − 17.025)	< 0.001	− 2.213 (− 3.457, − 0.969)	0.001	

Model a to m: Adjusted for potential confounding factors, including gender, age,BMI, married status, occupation, education level, smoking, alcohol drinking, history of hypertension, history of diabetes, history of hyperuricemia, history of gout, family history of gout, uric-acid-lowering drug intake.

*Having significant interactive effects (*P* < 0.05).

### The mediating effect of BMI between DASH diet score and SUA levels

**Figure 1:**
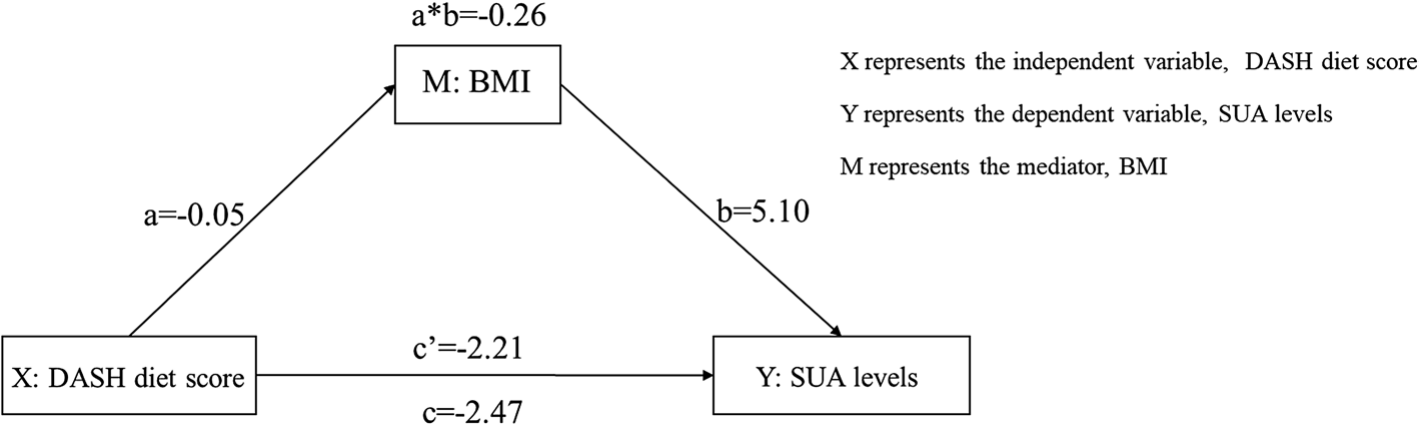
The pathway of the mediating effect of BMI between DASH diet score and SUA levels. c represents the total effect of the independent variable X on the dependent variable Y; a represents the relation between X and the mediator M; b represents the relation between M and Y adjusted for the effect of X; and c′ represents the effect of X on Y adjusted for the effect of M; The value of a*b denotes the mediating effect size.

According to the results of mediation analysis, after adjusting for gender, age, married status, occupation, education level, smoking, alcohol drinking, history of diabetes, history of hypertension, history of hyperuricemia, history of gout, family history of gout and uric-acid-lowering drug intake, the association between the DASH diet scores and SUA levels was partly mediated by BMI (− 0.26, Bootstrap 95% CI − 0.49, − 0.07), with 10.53% of total effect being mediated (Table 6, Fig. 1).

**Table 6:** The mediating effect of BMI between DASH diet score and SUA levels.

Effect	Effect Size	SEM	Bootstrap 95% CI		*P*	
Total effect	− 2.47	0.64	− 3.72	− 1.22	< 0.001	10.53^a^ (1.30, 19.76)^b^
Direct effect	− 2.21	0.63	− 3.44	− 0.97	< 0.001	
Indirect effect	− 0.26	0.11	− 0.49	− 0.07	−	
DASH diet score—BMI	− 0.05	0.02	− 0.09	− 0.01	0.011	
BMI—SUA levels	5.10	0.93	− 3.28	6.91	< 0.001	

^a^The proportion of mediating effect to total effect.

^b^95% CI.

## Discussion

As is revealed in this cross-sectional population-based study, greater adherence to the DASH diet was significantly associated with lower SUA levels of adult population in China, and the effect might be partly mediated by BMI, and after adjustment for potential confounding factors did not materially change the results.

It was demonstrated by this study that adopting the DASH diet might be beneficial to reduce SUA level. A randomized trial from the USA indicated that the DASH diet decreased the mean SUA by 0.25 mg/dL for the participants after an 8-week intervention period^38^. In addition, Olive et al. found that the DASH diet lowered the SUA levels by 0.8 and 1.0 mg/dL after 30 and 90 days respectively, among participants with baseline SUA ≥ 6 mg/dL^28^. These findings are consistent with the outcome of our study, which confirmed that adopting the DASH diet might be helpful in decreasing SUA levels.

Our study shown that the DASH diet was correlated negatively with the SUA levels among non-smoking, non-hypertension, and non-hyperuricemia participants. Specifically, participants who were non-smoking, non-hypertension, and non-hyperuricemia may be more self-disciplined and more likely to actively adopt and maintain a healthy diet than those who were smoking, hypertensive and hyperuricemia. Therefore, they were more likely to adopt the DASH diet, and the higher their tendency to maintain the DASH diet, the lower their blood uric acid level was.

Although previous studies have explored the association between the DASH diet and SUA levels in patients with hypertension and hyperuricemia, there are no consistent results^28,39,40^. We found that there was no relationship between the DASH diet and SUA levels in patients with hypertension and hyperuricemia. One possible explanation to this finding was that the number of samples with hypertension and hyperuricemia included in this research was small. Another possible reason was that the effect of the DASH diet on SUA levels is partially masked by its blood pressure-lowering effect. A study from Bangladesh showed that the SUA level was positively correlated with blood pressure^41^. Since the essential feature of the DASH diet proved to be the most effective dietary approach to reduce blood pressure, its effect on SUA levels could also be well documented. Therefore, whether the DASH diet has potential uric acid-lowering effects on patients with hypertension and hyperuricemia requires a further verification by longitudinal studies in China.

The results of this study discovered that the higher propensity of the individuals to adopt the DASH diet, the less likely they are to be overweight and obese, thus they were more likely to report lower SUA levels. A meta-analysis of randomized controlled clinical trials revealed that adherence to the DASH diet significantly decreases weight particularly for overweight and obese participants^29^.

The DASH diet advocates a high intake of fiber foods and a reduced intake of unsaturated fats. Therefore, limiting saturated fat and cholesterol intake may lead to lower concentrations of LDL-C and TC^42,43^, which further reduces obesity risk. Mechanistically, ingested fiber sequesters more cholesterol in the gut, thereby increasing its fecal elimination. Thus, the DASH diet reduces the transport of cholesterol and triglycerides to the circulation. Furthermore, fiber consumption is also known as a determinant of long-term weight loss, leading to improvements in obesity parameters due to its role in delayed gastric emptying and a certain reduction in macro-nutrient absorption^44,45^.

For adults, weight is a larger component of BMI. Because in adults, the height of the individual is relatively stable^46^, while the weight is affected by many aspects such as lifestyle, psychology, and environment, and changes greatly. The average age of the subjects in this study was around 40 years old, and their BMI was more susceptible to being influenced by weight, especially increased visceral fat.

Moreover, the high visceral fat content and strong stimulation of fatty acid synthesis is accompanied by hyper-metabolic conversion of ribose 5-phosphate in overweight /obese individuals, resulting in increased uric acid production^47^, as well as lower insulin sensitivity in overweight/obese individuals^48^, causing enhanced renal tubular re-absorption of uric acid and reduced uric acid excretion^49^. Epidemiological studies have also indicated that overweight and obesity are risk factors for elevated SUA^32,50^. These findings may provide new insights for that BMI play the role in generating the association between the DASH diet and SUA levels. Therefore, it is reasonable to conclude that the effect of the DASH diet on decreasing SUA levels may be further enhanced partly by its role in controlling BMI. Yet, it should be noticed that some studies revealed that there were reason-result relationships between elevated SUA levels and overweight/ obesity, which means that elevated SUA levels may also contribute to overweight /obesity^51^. Still, the chain of causation is largely unknown among the DASH diet, BMI, SUA levels in our research. The DASH diet causes the reduction of BMI, which leads to lower SUA levels in turn. Therefore, a longitudinal study design should be implemented in future to clarify their causal relationships, finding out whether the DASH diet causes BMI reduction which in turn leads to lower SUA levels, or the DASH diet causes BMI and SUA levels reduce parallelly, or the DASH diet causes changes in SUA levels and then affects BMI.

Clinical implications in this study we would like to emphasize are as follows. Firstly, for individuals with higher SUA levels, adhering to the DASH diet may help reduce their SUA levels; this strategy could be applied to health education on individuals for medical staff to carry out one-to-one guidance to strengthen individuals’ SUA management and reduce the incidence of elevated SUA levels. Secondly, adhering to the DASH diet is more effective in control SUA levels for individuals with higher BMI, especially overweight or obese adults. This finding further refines obesity management tools. Finally, adults with healthier lifestyles and better health status, such as non-smoking, non-hypertension and non-hyperuricemia individuals, are encouraged to adopt to the DASH diet to reduce their SUA levels.

Several strengths should be emphasized in this study. The DASH diet is recognized internationally as an effective way to prevent and control hypertension, however, there are few studies have explored the association between the DASH diet and SUA levels, which mainly be studied in western countries. Therefore, this study may supply more epidemiological evidence to evaluate the relevance between the DASH diet and SUA levels, and which was based on the population in China. There are still some limitations in this study. Firstly, our study is a cross-sectional study, so it is impossible to identify the causal relationship between the DASH diet and SUA levels, which requires a further verification by prospective studies. Secondly, due to the small number of participants with a history of hypertension, besides a certain selection bias cannot be excluded. Thirdly, the non-random sample may have raised the possibility of selection bias in the study, thus requiring the cautious extension of the study findings to the general population. Fourthly, most of information was relied on a self-reported food frequency questionnaire among participants, thus recall bias is hard to be avoided completely. Finally, the food frequency questionnaire used in this study didn’t comprehensively collect information related to dietary intake and there may be other dietary and non-dietary risk factors affecting SUA levels that may confound the results of the study.

In conclusion, the results of this cross-sectional study indicate that participants included in this study preferred to the DASH diet moderately. Participants in sample area, especially for non-smoking, non-hypertension and non-hyperuricemia individuals, adopting the DASH diet might be helpful in reducing SUA level, and the effect might be partly mediated by BMI.

## Supplementary Information

Supplementary Information.

## Data Availability

The data that support the findings of this study are available from the corresponding author, Prof. Zuxun Lu. Email: zuxunlu@hust.edu.cn
