# Supplementary material for: Association between adherence to the Dietary Approaches to Stop Hypertension diet and serum uric acid
Source: Sci Rep. 2023 Apr 18;13:6347. doi: 10.1038/s41598-023-31762-x (PMC10113210; doi:10.1038/s41598-023-31762-x)
Supplement: Supplementary file 1 — Supplementary Information. [file 41598_2023_31762_MOESM1_ESM.pdf]

Manuscript Title

Association between adherence to the Dietary Approaches to Stop Hypertension diet and serum uric acid

Author information

Jie Feng[1*], Yuchai Huang[2*], Haozhuo Wang[3], Chao Wang[4], Hongbin Xu[1], Pan Ke[1], Yan He[5], Qingfeng Tian[5], Shiyi Cao[1#], Zuxun Lu[1#]

Supplementary appendix 1.

STROBE Statement—Checklist of items that should be included in reports of ***cross-sectional studies***

	Item No	Recommendation	Page
**Title and abstract**	1	(*a*) Indicate the study's design with a commonly used term in the title or the abstract	1
		(*b*) Provide in the abstract an informative and balanced summary of what was done and what was found	3
**Introduction**			
Background/rationale	2	Explain the scientific background and rationale for the investigation being reported	4-5
Objectives	3	State specific objectives, including any prespecified hypotheses	4-5
**Methods**			
Study design	4	Present key elements of study design early in the paper	5-6
Setting	5	Describe the setting, locations, and relevant dates, including periods of recruitment, exposure, follow-up, and data collection	5-8
Participants	6	(*a*) Give the eligibility criteria, and the sources and methods of selection of participants	6-7
Variables	7	Clearly define all outcomes, exposures, predictors, potential confounders, and effect modifiers. Give diagnostic criteria, if applicable	7-9
Data sources/ measurement	8*	For each variable of interest, give sources of data and details of methods of assessment (measurement). Describe comparability of assessment methods if there is more than one group	7-9
Bias	9	Describe any efforts to address potential sources of bias	Not Applicable
Study size	10	Explain how the study size was arrived at	6
Quantitative variables	11	Explain how quantitative variables were handled in the analyses. If applicable, describe which groupings were chosen and why	7-9
Statistical methods	12	(*a*) Describe all statistical methods, including those used to control for confounding	10
		(*b*) Describe any methods used to examine subgroups and interactions	Not Applicable

		(*c*) Explain how missing data were addressed	Not Applicable
		(*d*) If applicable, describe analytical methods taking account of sampling strategy	Not Applicable
		(*e*) Describe any sensitivity analyses	8
**Results**			
Participants	13*	(a) Report numbers of individuals at each stage of study—eg numbers potentially eligible, examined for eligibility, confirmed eligible, included in the study, completing follow-up, and analysed	9
		(b) Give reasons for non-participation at each stage	Not Applicable
		(c) Consider use of a flow diagram	Not Applicable
Descriptive data	14*	(a) Give characteristics of study participants (eg demographic, clinical, social) and information on exposures and potential confounders	10-11
		(b) Indicate number of participants with missing data for each variable of interest	Not Applicable
Outcome data	15*	Report numbers of outcome events or summary measures	10-12
Main results	16	(*a*) Give unadjusted estimates and, if applicable, confounder-adjusted estimates and their precision (eg, 95% confidence interval). Make clear which confounders were adjusted for and why they were included	10-12
		(*b*) Report category boundaries when continuous variables were categorized	10-12
		(*c*) If relevant, consider translating estimates of relative risk into absolute risk for a meaningful time period	Not Applicable
Other analyses	17	Report other analyses done—eg analyses of subgroups and interactions, and sensitivity analyses	11-12
**Discussion**			
Key results	18	Summarise key results with reference to study objectives	13
Limitations	19	Discuss limitations of the study, taking into account sources of potential bias or imprecision. Discuss both direction and magnitude of any potential bias	16-17
Interpretation	20	Give a cautious overall interpretation of results considering objectives, limitations, multiplicity of analyses, results from similar studies, and other relevant evidence	13-17
Generalisability	21	Discuss the generalisability (external validity) of the study results	16-17
**Other information**			
Funding	22	Give the source of funding and the role of the funders for the present study and, if applicable, for the original study on which the present article is based	21

*Give information separately for exposed and unexposed groups.

Supplementary appendix 2.

Community Resident Hyperuricemia Risk Assessment Questionnaire

Instructions

There are Four Parts in the questionnaire: Demographic characteristics,Lifestyles, History of diseases and family history of diseases, and Dietary intake.

The answers are privileged information.

There is no right or wrong answer,because it is essential to express your real feelings.

Please tick (√) appropriate one or or fill in “_____” directly.

Part 1: Demographic characteristics

1.Gender

□Male

□Female

2.Age

______ year old

3.Height

_______ cm (keep one decimal place)

4.Weight

_______ kg (keep one decimal place)

5.Marital status

□Married

□Not married

6.Education level

□College degree less

□College degree or higher

7.Occupation

□Medical staffs

□Not medical staffs

Part 2: Lifestyles

1.Smoking

□Yes

□No

2.Alcohol drinking

□Yes

□No

3.Uric-acid-lowering drug intake

□Yes

□No

Part 3: History of diseases and family history of diseases

1.History of hypertension

□Yes

□No

2.History of diabetes
□Yes

□No

3.History of hyperuricemia
□Yes

□No

4.History of gout
□Yes

□No

5.Family History of gout
□Yes

□No

Part 4: Dietary intake

The frequency of food consumption in the past six months.Please tick (√) appropriate one.

Food item	less than once per month	1~3 times per month	once per week	1~3 times per week	4~6 times per week	once per day	2~3 times per day	4~5 times per day	more than 5 times per day
refined grain									
coarse grain									
fresh fruits									
fruit products									
fresh vegetables									
pickles									
whole milk									
low-fat milk									
red meats									
white meats									
animal offal									
seafood									
processed meats									
legumes and their products									
nuts									
sweet drinks									
desserts									
smoked products									

End of form. Thank you again for your support and help with this study.
I wish you a happy life. Good luck with your work.
